# Cervical, anal and oral HPV detection and HPV type concordance among women referred for colposcopy

**DOI:** 10.1186/s13027-020-00287-7

**Published:** 2020-04-15

**Authors:** Maria Nasioutziki, Kimon Chatzistamatiou, Panagiotis-Dimitrios Loufopoulos, Eleftherios Vavoulidis, Nikolaos Tsampazis, George-Chrysostomos Pratilas, Anastasios Liberis, Vasiliki Karpa, Evanggelos Parcharidis, Angelos Daniilidis, Konstantinos Spanos, Konstantinos Dinas

**Affiliations:** 1 2nd Obstetrics & Gynaecology Department, Molecular & Morphological Clinical Cytopathology Laboratory, Hippokration General Hospital, Medical Faculty, School of Health Sciences, Aristotle University of Thessaloniki, Thessaloniki, Greece; 2 1st Department of General Surgery, Anal & Colorectal Clinic, Papageorgiou General Hospital, Medical Faculty, School of Health Sciences, Aristotle University of Thessaloniki, Thessaloniki, Greece; 3 Stomatology Clinic, School of Dentistry, Aristotle University of Thessaloniki, Thessaloniki, Greece

**Keywords:** Anal, cervical, oral HPV concordance, Anal HPV-detection, Cervical HPV-detection, Human papilloma virus, Oral HPV-detection

## Abstract

**Background:**

Infection with human papillomaviruses (HPVs) can cause benign and malignant tumours in the anogenital tract and the oropharynx both in men and women. The aim of the presented study was to investigate cervical, anal, and oral HPV-detection rates among women referred to colposcopy for abnormal Cervical Cancer (CaCx) screening results and assess the concordance of HPV-types among these anatomical sites.

**Methods:**

Women referred to colposcopy at a single centre due to abnormal cytology, conducted for CaCx screening, were subjected to cervical Liquid-based Cytology (LBC) smear testing, anal and oral sampling. Routine colposcopy consisted in multiple biopsies and/or Endocervical Curettage (ECC). HPV-detection was performed by PCR genotyping in all three anatomical sites. In high-risk (hr) HPV-DNA positive samples either from anal canal or oral cavity, anal LBC cytology and anoscopy were performed, or oral cavity examination respectively. Descriptive statistics was used for the analysis of HPV-detection rates and phi-coefficient for the determination of HPV-positivity concordance between the anatomical sites.

**Results:**

Out of 118 referred women, hr. HPV-DNA was detected in 65 (55.1%), 64 (54.2%) and 3 (2.5%) at cervix, anal canal and oral cavity respectively while low-risk HPV-DNA was detected in 14 (11.9%) and 11 (9.3%) at cervix and anal canal respectively. The phi-coefficient for cervix/anal canal was 0.392 for HPV16, 0.658 for HPV31, 0.758 for HPV33, − 0.12 for HPV45, 0.415 for HPV52 and 0.473 for HPV58. All values were statistically significant (*p* < 0.001).

**Conclusions:**

The results suggest that most HPV-types, high-risk and low-risk, detected in the cervix of women with prevalent cervical dysplasia, correlate with the ones detected in their anal canal. This particularly applies for the HPV-types included in the nonavalent HPV-vaccine (HPVs 6/11/16/18/31/33/45/52/58).

## Background

Persistent infection with high-risk human papillomaviruses (HPVs) is necessary, but not sufficient, for the development of Cervical Cancer (CaCx) [1–3]. HPV has been detected in other anatomical sites like the anus and the oral cavity and has been associated to some extent to anal and oropharyngeal cancer respectively [4–7].

Anal HPV-related cancer is strongly associated with the immunosuppression secondary to HIV-infection and associated with a history of anal-receptive intercourse; however, autoinoculation of vulvar warts to the perianal skin can occur. Clinically, anal HPV-infection can be either asymptomatic or present as benign warts (*condylomata acuminata*), dysplastic lesions (anal intraepithelial neoplasia-AIN), or a combination of both [8, 9].

Anal HPV-prevalence is higher in men who have sex with men (MSM) (58.8%), for any HPV-type, whereas it is 30.7% for women [10]. Moreover, it has been reported that anal HPV-infection is more common in men or women who already have a genital HPV-infection suggesting HPV-type concordance in genitalia and anus [11–14].

Less is known concerning oral HPV-infection, its prevalence and its implications including the involvement of HPV in oropharyngeal carcinogenesis [7, 15]. HPV-prevalence in the oral cavity of women is 4.4%, similar to that of men (4.6%), however it has been reported to be higher for high-risk (hr) groups [16]. Also, in a systematic review of studies reporting on HPV-detection rates in oral carcinomas and potentially malignant oral-disorders compared with healthy controls, it was shown that pooled HPV-DNA detection rate was higher in case of the former compared to controls [17].

Prophylactic HPV-vaccines have been implemented in the national vaccination programs of many countries for the primary prevention of CaCx and other HPV-associated diseases [18]. Bivalent- (for HPVs 16/18), quadrivalent- (for HPVs 6/11/16/18) and, nonavalent- vaccines (for HPVs 6/11/16/18/31/33/45/52/58) are considered effective and able to reduce most HPV-related cancers [19].

Women who already have a cervical HPV-infection and especially women who have a precancerous cervical intraepithelial lesion (CIN) are considered to be susceptible to HPV-infections in other anatomical sites [20]. To investigate cervical/anal/oral HPV-detection rates among such a group of women, as well as to assess the concordance of different HPV-types among these anatomical sites, we conducted the presented analysis on women attending a colposcopy clinic who, most likely, have already a cervical HPV-infection, either transient or productive.

## Materials & methods

### Study setting, participants & sampling

The presented analysis was conducted on samples collected from 118 women referred to the Colposcopy/Cervical Pathology Clinic of the 2nd Department of Obstetrics/Gynaecology (Hippokration Hospital, Medical Faculty Aristotle University of Thessaloniki (AUTh), Greece) for either an abnormal Pap-test screening result or for follow-up testing after surgical treatment for high-grade CIN between January 2015 and November 2016. Exclusion criteria had been pregnancy, prior total hysterectomy due to various reasons, and prior chemo-radiation treatment. All women with anal hr-HPV positivity where referred to the Anal/Colorectal Clinic of the 1st Department of General Surgery, Medical Faculty AUTh, for anoscopy. Women with oral hr-HPV positivity were referred for oral examination to the Stomatology Clinic, School of Dentistry, Faculty of Health Sciences AUTh.

Each woman was subjected to cervical/anal/oral sampling. All samples were processed at the Molecular Cytopathology Laboratory of the 2nd Department of Obstetrics/Gynaecology. Ectocervical and endocervical sampling was done using a plastic Ayre’s Spatula and a Cytobrush® (CooperSurgical, USA) respectively, and anal sampling was done using the Anex® Brush (Rovers Medical Devices, Netherlands). After collection each device was directly immersed in a vial containing PreservCyt® collection fluid (Hologic, USA), according to manufacturer’s instructions. Oral samples were collected by an oral rinse with 15 mL Listerine® for 30 s [21, 22] in sterile vials. All samples were kept between 2 and 8 °C, for up to 2 days prior to testing.

### Cytology testing

Cytology testing was performed on cervical/anal samples using Liquid-Based Cytology (LBC) (ThinPrep®, Hologic, USA), prior to molecular testing. Cytology results were classified according to Bethesda 2014 classification [23]. ASCUS or worse (ASCUS+) results for cervical or anal samples were considered positive and required further action. Cervical cytology is routinely performed on women referred to the Colposcopy Clinic whereas anal cytology was performed in conjunction with HPV-DNA testing to assess the need for anoscopy referral (although in our study all anal hr-HPV positive women were referred).

### HPV DNA genotyping

DNA extraction/purification was performed using CLART-HPV2 Extraction/Purification kit (Genomica, Spain). CLART-HPV2 detect infections and coinfections of 35 most clinically hr- (hr) and low-risk (lr) HPV-types (6/11/16/18/26/31/33/35/39/40/42/43/44/45/51/52/53/54/56/58/59/61/62/66/68/70/71/72/73/81/82/83/84/85/89). Protocol was carried out according to manufacturer’s instructions. One ml of PreservCyt solution was used for HPV-DNA extraction with purifying columns and 5 μl of that was used for PCR-Amplification. Prior to visualization, PCR-products were denaturated at 95 °C for 10 min. Visualization was performed using 5 μl denaturated product on CLART-microarray. Analysis and interpretation of results was automatically performed by GENOMICA’s reader (CAR®).

### Colposcopy and histological examination

Colposcopic evaluation was performed according to 2011 colposcopic terminology of International Federation for Cervical Pathology and Colposcopy [24]. Routine colposcopic examination consisted in multiple biopsies and/or Endocervical Curettage (ECC) (in case of a type-2 or type-3 transformation zone). Histology results could be within normal limits, Low-grade Squamous Intraepithelial Lesion (LSIL), High-grade Squamous Intraepithelial Lesion (HSIL) or cancer, according to the Lower Anogenital Squamous Terminology (LAST) [25]. Histology examination was conducted by pathologists, at Histopathology Department Hippokration Hospital, Thessaloniki, Greece. Pathological findings (SIL of any degree) were re-assessed by an independent specialized pathologist and a third pathologist in case of disagreement.

### Anoscopy

High-resolution anoscopy (HRA) was performed at the Anal/Colorectal Outpatient Clinic of the 1st Department of General Surgery, Papageorgiou Hospital, AUTh, Thessaloniki, Greece. An anoscope was inserted and a colposcope was used to examine the squamocolumnar junction, the anal canal including the transformation zone and the perianal skin in a systematic manner. After examination with acetic acid (3% or 5%), and subsequent application of Lugol’s iodine solution, a decision was made regarding the anoscopic impression, which could be either within normal limits, or indicative of a LSIL, HSIL or cancer. In case of an abnormal anoscopic impression a biopsy was taken [26]. If HRA was not available, simple anoscopy was performed (providing less information regarding lesion identification and characterization). Prior to anoscopy, anal inspection and digital rectal examination was performed in all patients.

### Oral examination

Oral visualization was conducted using an adequate light source in a systematic way. All mucosal surfaces were examined and lesions were recorded on a diagram. The lips were inspected first, followed consecutively by the labial mucosa, buccal mucosa, floor of the mouth, ventrum of the tongue, dorsal surface of the tongue, hard and soft palates, gingivae and teeth [27].

### Statistical analysis

The main purpose of the analysis was to assess the concordance of different HPV-types among different anatomical sites in women. Phi-coefficient was used to determine HPV positivity concordance between cervical/anal, cervical/oral, anal/oral and was calculated for each HPV-type or HPV-types group concerning these anatomical site pairs. The HPV-type groups were hr-HPV, lr-HPV and the nonavalent HPV vaccine types group (HPVs 6/11/16/18/31/33/45/52/58). Phi-coefficient can range from −1 (strong negative correlation) to +1 (strong positive correlation). Values near zero indicate no correlation. For each phi-value calculated we report the respective *p*-value. The level of significance was set to 0.05.

Smoking habits were assessed by Smoking Intensity Index (SII), a new variable created as below: Number (cigarette/day) × 365 × Number (years) ÷ 1000 [28].

All analyses were performed using statistical software SPSS Statistics 22.0.

## Results

### Demographics

The age of the recruited women ranged from 21 to 61 years (mean age 38.6 years). Table 1 shows demographic characteristics. Of the women recruited 50.8% reported higher education and 41.5% had no children. Most of them were of reproductive age since only 14.4% reported being in menopause. Combined oral contraceptive use was reported by 8.5% whereas 48.3% reported no contraceptive method. Moreover, 57.6% reported being current smokers and 6.8% reported being former smokers (meaning they had stopped smoking at least 1 year prior to recruitment). Among current smokers 45.5% were light-smokers (SII < 50), 20.6% were medium- (SII 50–100) and 33.9% were heavy-smokers (SII > 100). Most of them (63.6%) reported more than four sexual partners and mean age of first sexual intercourse was 18.4 years (range: 14 to 35).

**Table 1:** Demographic characteristics of the study population

Demographic characteristic	*N* = 118
Age (years)	
Mean (sd)	38.6 (10.2)
Median (range)	37 (21–61)
Educational level	
6 y (primary)	11 (9.3%)
12 y (secondary)	38 (32.2%)
Technological institute	29 (24.6%)
University	26 (22%)
Post-grad	5 (4.2%)
Children	
No	49 (41.5%)
1	22 (18.6%)
2	38 (32.2%)
3	4 (3.4%)
4	4 (3.4%)
Menopause	
No	101 (85.6%)
Yes	17 (14.4%)
Contraception	
No	57 (48.3%)
COC	10 (8.5%)
IUD	3 (2.5%)
Male condom	38 (32.2%)
Interrupted intercourse	5 (4.2%)
HPV vaccination	
No	87 (73.7%
Quadrivalent	14 (11.9%)
Bivalent	4 (3.4%)
Smoking history	
No	32 (27.1%)
Yes	68 (57.6%)
Ex-smoker	8 (6.8%)
SII	
<50	31 (27.1%)
50–100	14 (11.9%)
>100	23 (19.5%)
Number of sexual partners	
1	10 (8.5%)
2	10 (8.5%)
3	19 (16.1%)
≥4	75 (63.6%)
Age of first sexual intercourse	
Mean (sd)	18.4 (2.9%)
Median (range)	18.0 (14–35)

*COC* combined oral contraceptives, *IUD* intrauterine device, *SII* smoking intensity index

### HPV-prevalence

As expected, due to the characteristics of the referral population in this study, cervical HPV-prevalence was high. In particular, hr-HPV prevalence was 55.1% (Table 2). Among hr-HPVs the most common was HPV16 (22.0%), followed by HPV31 (13.6%), HPVs 51/52 (each 7.6%), HPVs 33/53 (each 5.9%), and HPV58 (5.1%). The remaining hr-HPVs presented prevalence below 2.5%. Regarding lr-HPVs, the overall prevalence was 11.9%. Among lr-HPVs, the most commonly detected type was HPV42 (5.9%), followed by HPV61 (4.2%) with the rest lr-HPVs being less than 3.4% of the cases.

**Table 2:** Cervical, anal and oral HPV prevalence for the study population (percentages refer to the total of each age group)

	Cervical [*n* = 118 (100.0%)]	Anal [*n* = 118 (100.0%)]	Oral [*n* = 118 (100.0%)]
HRHPV	65 (55.1%)	64 (54.2%)	3 (2.5%)
LRHPV	14 (11.9%)	11 (9.3%)	0 (0.0%)
HR+LRHPV	9 (7.6%)	23 (19.5%)	0 (0.0%)
HPV16/18	26 (22.0%)	16 (13.5%)	2 (1.7%)
HRHPV16	26 (22.0%)	11 (9.3%)	2 (1.7%)
HRHPV18	0 (0.0%)	5 (4.2%)	0 (0.0%)
HRHPV31	16 (13.6%)	18 (15.3%)	0 (0.0%)
HRHPV33	7 (5.9%)	6 (5.1%)	0 (0.0%)
HRHPV35	3 (2.5%)	3 (2.5%)	0 (0.0%)
HRHPV39	2 (1.7%)	0 (0.0%)	0 (0.0%)
HRHPV45	2 (1.7%)	1 (0.8%)	0 (0.0%)
HRHPV51	9 (7.6%)	12 (10.2%)	0 (0.0%)
HRHPV52	9 (7.6%)	5 (4.2%)	0 (0.0%)
HRHPV53	7 (5.9%)	20 (16.9%)	1 (0.8%)
HRHPV56	2 (1.7%)	1 (0.8%)	0 (0.0%)
HRHPV58	6 (5.1%)	6 (5.1%)	0 (0.0%)
HRHPV59	2 (1.7%)	4 (3.4%)	0 (0.0%)
HRHPV66	1 (0.8%)	8 (6.8%)	0 (0.0%)
HRHPV68	0 (0.0%)	0 (0.0%)	0 (0.0%)
HRHPV70	0 (0.0%)	7 (5.9%)	0 (0.0%)
HRHPV73	0 (0.0%)	1 (0.8%)	0 (0.0%)
HRHPV82	2 (1.7%)	–	–
LRHPV6	4 (3.4%)	10 (8.5%)	0 (0.0%)
LRHPV11	1 (0.8%)	1 (0.8%)	0 (0.0%)
LRHPV40	1 (0.8%)	4 (3.4%)	0 (0.0%)
LRHPV42	7 (5.9%)	5 (4.2%)	0 (0.0%)
LRHPV44	0 (0.0%)	2 (1.7%)	0 (0.0%)
LRHPV54	2 (1.7%)	2 (1.7%)	0 (0.0%)
LRHPV61	5 (4.2%)	5 (4.2%)	0 (0.0%)
LRHPV62	1 (0.8%)	3 (2.5%)	0 (0.0%)
LRHPV65	0 (0.0%)	0 (0.0%)	0 (0.0%)
LRHPV71	0 (0.0%)	1 (0.8%)	0 (0.0%)
LRHPV81	3 (2.5%)	5 (4.2%)	0 (0.0%)
LRHPV83	0 (0.0%)	1 (0.8%)	0 (0.0%)
LRHPV84	1 (0.8%)	3 (2.5%)	0 (0.0%)

*HRHPV* high-risk human papillomavirus, *LRHPV* low-risk human papillomavirus, *9HPV* human Papillomaviruses targeted by the nonavalent HPV vaccine

Anal HPV-prevalence presented similar trend to the cervical one with minor differences. The overall anal hr-HPV prevalence was 54.2%. The most common hr-HPV type was HPV53 (16.9%), followed by HPV31 (15.3%), HPV51 (10.2%), HPV16 (9.3%), HPV66 (6.8%), HPV70 (5.9%), HPVs 33/58 (each 5.1%). Remaining hr-HPVs presented prevalence below 4.2%. Lr-HPV-types were detected in 9.3% of the cases; the most common lr-HPV was HPV6 (8.5%), followed by HPVs 42/61/81 (4.2% each), HPV40 (3.4%). The prevalence of the remaining lr-HPVs was less than 2.5% for each. Out of the 64 women recruited for the current analysis positive for hr-HPV types and negative for intraepithelial lesion or malignancy (NILM) (42.2%) or LSIL (57.8%) anal cytology, only 15 women underwent anoscopy (despite their referral) with no pathological or suspicious lesions detected.

Oral HPV was detected much less frequently than cervical and anal. The overall hr-HPV and lr-HPV prevalence was 2.5 and 0.0% respectively. The only hr-HPV types detected were HPV16 (1.7%) and HPV53 (0.8%). No lesions were detected in the oral cavity of the women referred to oral examination.

A hr-HPV and lr-HPV co-infection was documented in 7.6, 19.5 and 0.0% concerning cervical/anal/oral samples respectively (Table 2). The prevalence of at least one of the nine HPV-types included in nonavalent HPV vaccine was 47.5, 44.1, and 1.7% for cervix/anus/oral cavity respectively (Table 3). The prevalence of these types fluctuated with age, being highest for age group 36–40 (75.0% cervical, 70.0% anal, 5.0% oral samples), lowest for women aged 46–50 (30.0% cervical samples) and below 25 and 51–55 (14.1% anal samples). A similar trend was observed for hr-HPVs concerning different age groups. Briefly, cervical hr-HPV detection was highest for women aged 36–40 (80.0%) and lowest for women 46–50 (30.0%), anal hr-HPV was highest for women 26–30 (76.9%) and lowest for women below 25 (28.6%). Low-risk types are more commonly detected in the cervix below age 25 compared to older age (28.6% vs 23.1% or less), however, concerning the anal canal, it seems that younger women are less likely to have lr-HPV infection compared to older ones (21.4% vs 21.7 at least) (Table 3).

**Table 3:** HPV type prevalence according to age group and anatomical site

	< 25 (%)*	26–30 (%)*	31–35 (%)*	36–40 (%)*	41–45 (%)*	46–50 (%)*	51–55 (%)*	> 56 (%)*	Total (%)
HRHPV (cervical)	64.3	61.5	45.5	80.0	52.2	30	42.9	44.4	55.1
LRHPV (cervical)	28.6	23.1	18.2	15.0	21.7	10.0	14.3	22.2	19.5
HRHPV (anal)	28.6	76.9	50.0	75.0	52.2	50.0	57.1	33.3	54.2
LRHPV (anal)	21.4	46.2	27.3	30.0	21.7	30.0	28.6	33.3	28.8
HRHPV (oral)	0.0	0.0	0.0	5.0	8.7	0.0	0.0	0.0	2.5
LRHPV (oral)	0.0	0.0	0.0	0.0	0.0	0.0	0.0	0.0	0.0
9HPV (cervical)	42.9	46.2	40.9	75.0	47.8	30.0	42.9	33.3	47.5
9HPV (anal)	14.3	69.2	36.4	70.0	52.2	40.0	14.3	22.2	44.1
9HPV (oral)	0.0	0.0	0.0	5.0	4.3	0.0	0.0	0.0	1.7

*HRHPV* high-risk human papillomavirus, *LRHPV* low-risk human papillomavirus, *9HPV* human papillomaviruses targeted by the nonavalent HPV vaccine

^*^Percentages refer to the total of each age group

### HPV-detection concordance among different anatomical sites

Most of HPV-types detected in cervical samples were detected also in anal samples, and for the majority of them there has been a statistically significant correlation. The phi-coefficient, regarding the cervical/anal anatomical site pair, for hr-HPVs was 0.402 (*p* < 0.001). For cervical/oral and anal/oral phi-coefficient values were −0.071 and 0.04 with *p*-values 0.443 and 0.662 respectively, indicating no correlation for the detection of these HPV-types in cervix or anus compared to oral cavity. HPV16-detection yielded similar results. Phi-coefficient values for this type were 0.392 (*p* < 0.001), 0.088 (*p* = 0.341) and −0.042 (*p* = 0.646) for cervix compared to anus or oral cavity and for anus compared to oral cavity respectively.

Another HPV-type which yielded correlation results for the three anatomical sites was HPV53. For the cervical/anal pair phi-coefficient was 0.365 (*p* < 0.001), for cervical/oral − 0.023 (*p* = 0.8), and for anal/oral −0.041 (*p* = 0.658). All remaining hr-HPV types yielded results only for the cervical/anal anatomical site pair. The strongest correlations, with a phi-coefficient above 0.7 were noted for HPV33 (phi = 0.758, *p* < 0.001), HPV35 (phi = 1.0, *p* < 0.001), HPV51 (phi = 0.748, *p* < 0.001), and HPV56 (phi = 0.704, *p* < 0.001). Regarding the nonavalent-vaccine HPVs, correlation was significant for the cervical/anal samples (phi = 0.353, *p* < 0.001), and cervical/oral samples (phi = 0.007, *p* = 0.942) (Table 4).

**Table 4:** Phi coefficient regarding hr- & lr-HPV DNA detection among different anatomical sites

	Cervical/anal Phi (95%CI, p)	Cervical/oral Phi (p)	Anal/oral Phi (p)
HRHPV	0.402 (< 0.001)	−0.071 (0.443)	0.04 (0.662)
LRHPV	0.443 (< 0.001)	–	
HRHPV16	0.392 (< 0.001)	0.088 (0.341)	−0.042 (0.646)
HRHPV18	–	–	–
HRHPV31	0.658 (< 0.001)	–	–
HRHPV33	0.758 (< 0.001)	–	–
HRHPV35	1.0 (< 0.001)	–	–
HRHPV39	–	–	–
HRHPV45	−0.12 (0.895)	–	–
HRHPV51	0.748 (< 0.001)	–	–
HRHPV52	0.415 (< 0.001)	–	–
HRHPV53	0.365 (< 0.001)	−0.023 (0.8)	−0.041 (0.658)
HRHPV56	0.704 (< 0.001)	–	–
HRHPV58	0.473 (< 0.001)	–	–
HRHPV59	0.338 (< 0.001)	–	–
HRHPV66	0.368 (< 0.001)	–	–
HRHPV68	–	–	–
HRHPV70	–	–	–
HRHPV73	–	–	–
HRHPV82	–	–	–
LRHPV6	0.447 (< 0.001)	–	–
LRHPV11	1.0 (< 0.001)	–	–
LRHPV40	0.494 (< 0.001)	–	–
LRHPV42	0.481 (< 0.001)	–	–
LRHPV44	–	–	–
LRHPV54	–	–	–
LRHPV61	0.373 (< 0.001)	–	–
LRHPV62	−0.015 (0.871)	–	–
LRHPV65	–	–	–
LRHPV71	–	–	–
LRHPV81	0.501 (< 0.001)	–	–
LRHPV83	–	–	–
LRHPV84	−0.015 (0.871)	–	–
Nonavalent HPV vaccine types	0.353 (< 0.001)	0.007 (0.942)	

Low-risk HPV-types were positively correlated among cervical and anal samples (phi = 0.443, *p* < 0.001). Specifically, the strongest correlation was reported for HPV11 (phi = 1.0, *p* < 0.001), no correlation was reported for HPVs 62 (phi = − 0.015, *p* = 0.871) and 84 (phi = − 0.015, *p* = 0.871). The rest lr-HPVs presented weak (0.3 < phi < 0.7) correlations (Table 4).

## Discussion

In the recent years, there has been an increasing interest in HPV-infection concerning anatomical areas other than the cervix. It has been hypothesized that HPV could be a carcinogenic factor for anal as well as oropharyngeal cancer, apart from cervical, for which it is known to be a necessary condition, since hr-HPV is detected in 99.7% of all cervical cancer cases worldwide [1].

Anal cancer is the second most related to HPV cancer after cervical, since HPV DNA has been reported to be present in 88% of anal cancer cases, with HPV16 being the predominant type [6, 29]. Moreover, the incidence of anal cancer has been increasing during the past decades particularly for high-risk groups, both for males and females [30].

Epidemiological features of anal cancer share similarities with the ones for anal HPV-infection. MSM consist a high-risk group for both conditions and for this group HPV-prevalence is about 58.8% for any HPV-type, 29.1% for hr-HPVs and 33.3% for lr-HPVs. Concerning the most common and important HPV-type, HPV16, its prevalence for MSM is 11.4%. As far as men who have sex with women are concerned, anal HPV-prevalence is 14.2% for any HPV, 5.5% for hr-HPVs, 9.0% for lr-HPVs and 1.6% for HPV16, whereas regarding women, the respective values are 30.7%, 13.1%, 17.1%, and 4.3% [10]. Therefore, it seems that women from the general population, are more likely to have an anal HPV-infection than men (with the exception of MSM). Also, women seem to be affected more than men by anal cancer which presents 50% higher incidence in females than males [31–33].

Women with HPV-related pathology of the genitalia, namely the vulva, the vagina and particularly the cervix tend to have anal HPV-infections more frequently. Anal HPV-prevalence for these women ranges from 23% to 86% compared to 5–22% for women with no known HPV-related pathology as indicated by a systematic review on female anal HPV-infection [12]. Particularly, regarding women with cervical dysplasia or with history of cervical cancer, there are studies indicating a high anal HPV-prevalence compared to women without any [34–39]. In our analysis of a colposcopy referral population, we found an anal HPV-prevalence of 54.2% for hr-HPVs, with HPV53, being the most frequently detected type, followed by HPV31, HPV51 and HPV16. Other studies report different order of the most frequent HPVs detected in the anal canal, however, HPVs 16, 53, and 51 have been reported to be in the first five most frequent types [40].

The higher anal HPV-prevalence in individuals with genital HPV-infection has been attributed to self-inoculation. This phenomenon has been shown in men in a sub-analysis of the HIM study reported by Pamnani et al. [41], and it is supported by the quite common finding of the detection of the same HPV types in the anal canal and the genitalia of women and men who have sex with women [11]. The current analysis has shown a statistically significant correlation among the HPV types detected in the anus and the ones detected in the cervix of women referred for colposcopy and this applied to most of the HPV types examined apart from three exceptions, namely hr-HPV45, lr-HPV62 and lr-HPV84. However, the limited number of the participants (*n* = 118) cannot provide the analysis with the power necessary for robust conclusions concerning the interpretation of these associations.

Genital HPV-infection prevalence in women decreases with increasing age [42], and the same phenomenon has been noted concerning anal HPV-detection [40, 43]. We could not demonstrate this decline since the population tested in the current analysis was a referral one. This is the reason that even for cervical HPV-infection, for which the age-related pattern is well established, the highest hr-HPV prevalence was presented by women aged 36–40, and not by women younger than 25. Moreover, anal HPV-detection rate fluctuated, more or less, in a similar way to the cervical one, concerning different age groups, with the exception of two age groups, namely women younger than 25 who presented a cervical hr-HPV-detection rate of 64.3% and an anal one of 28.6%, and for women 46–50, for whom the respective values were 30 and 50% respectively.

In the recent years, there has been significant research on HPV-infection in anatomical sites other than the anogenital area. In particular, there has been an increasing interest in the detection of HPV in the oral cavity and the possible relation of HPV-infection to oropharyngeal cancer. This concerns both sexes but mostly men since this type of cancer is quite more frequent in men than women [44]. However, a systematic review has shown that oral HPV-infection is similar to both sexes, 4.6% in men and 4.4% in women [16]. We showed that oral HPV-prevalence was 2.5% for hr-HPV types in a population at increased risk for HPV-infections. This figure is low compared to the ones reported in the literature for such a population. A meta-analysis on oral HPV-detection rate in women with a cervical HPV-infection yielded a pooled prevalence of 18.1%, and a rate of HPV-type concordance of 27%, which was statistically significant meaning that they found an association between oral and cervical HPV-infection [45]. HPV-type concordance was not documented in our analysis between the cervix or the anus and the oral cavity, however, this is not a robust result since the number of women tested positive for oral HPV was very limited.

## Conclusions

As a conclusion, our results suggest that most HPV-types, high and low-risk, detected in the cervix of women with prevalent cervical dysplasia, correlate with the ones detected in the anal canal of these women. This particularly applies for the HPV-types related to the nonavalent HPV vaccine (HPVs 6/11/16/18/31/33/45/52/58), implying that this vaccine could be used as the “first line of protection”, apart from cervical cancer, for anal cancer as well.

## Data Availability

All data generated or analyzed during this study are included in this published article.

## Abbreviations

HPV: Human Papilloma Virus
hr.: High-risk
lr: Low-risk
MSM: Men who have sex with men
HRA: High-resolution anoscopy
LBC: Liquid-based Cytology
ECC: Endocervical Curettage
